# Association between hyperuricemia and long-term mortality in patients with hypertension: results from the NHANES 2001–2018

**DOI:** 10.3389/fcvm.2024.1306026

**Published:** 2024-02-06

**Authors:** Yufeng Yin, Erye Zhou, Jian Wu

**Affiliations:** Department of Rheumatology, The First Affiliated Hospital of Soochow University, Suzhou, Jiangsu, China

**Keywords:** hyperuricemia (HUA), mortality, hypertension, cardiovascular diseases, NHANES

## Abstract

**Objective:**

The prevalence of hyperuricemia and hypertension is steadily increasing, and these conditions often share common risk factors. This study aimed to investigate the association among hyperuricemia, hypertension, and all-cause mortality in a nationally representative U.S. population.

**Methods:**

Data for 38,644 participants were obtained from the National Health and Nutrition Examination Survey (NHANES) 2001–2018. Hyperuricemia was defined as a serum urate concentration >420 μmol/L in men and >360 μmol/L in women. Information regarding death outcomes was obtained through the National Death Index (NDI). Multivariate logistic regression, Cox proportional hazards models, and restricted cubic spline (RCS) analyses were used to evaluate the association between hyperuricemia and hypertension in all included participants, as well as long-term mortality in patients with hypertension.

**Results:**

Among all participants, 6,956 (18.0%) had hyperuricemia, while 31,688 (82.0%) had nonhyperuricemia. According to the adjusted models, hyperuricemia was more strongly associated with hypertension (OR 2.04) than was non-hyperuricemia. During the median follow-up period of 78 months, both hyperuricemia (HR 1.48, 1.95) and hypertension (HR 1.42, 1.69) independently associated with the increased risk of all-cause mortality and cardiovascular mortality, respectively, with the highest risk observed in those with both conditions (HR 1.87, 2.82). RCS analyses revealed nonlinear J-shaped (for hypertension) and U-shaped (for both all-cause and cardiovascular mortality) relationships with serum urate levels.

**Conclusions:**

Hyperuricemia is associated with an elevated risk of developing hypertension compared to non-hyperuricemia. Among patients with hypertension, those with hyperuricemia are more likely to experience all-cause and cardiovascular mortality during long-term follow-up.

## 1 Introduction

Serum urate is a metabolic byproduct of purine breakdown. When concentrations exceed the limit of serum saturation, known as hyperuricemia, crystals of monosodium urate may precipitate in joints and tissues, causing inflammation and gouty arthritis, which affects 8.3 million Americans (1). While it is important to note that not all individuals with hyperuricemia experience symptomatic complications (2), epidemiological evidence underscores its correlation with an increased risk of metabolic syndrome, cardiovascular diseases, chronic renal disease, and premature mortality (3, 4). Hypertension has a profound global impact, affecting approximately 1.13 billion individuals worldwide (5). According to the World Health Organization (WHO), this condition is a contributing factor to 13% of all deaths worldwide (5). The development of hypertension is attributed to a multifaceted and intricate interplay of factors. Over recent decades, our understanding of this condition has grown, with increasing recognition of the various elements that contribute to its onset and progression (5).

Hyperuricemia often precedes the development of systemic hypertension, and multiple prospective studies have confirmed hyperuricemia as an independent risk factor for future hypertension, as well as poorly controlled blood pressure (4, 6–8). A 1 mg/dl increase in the serum urate concentration above the normal level has been associated with an 8%–13% increase in the adjusted risk of new-onset hypertension (9). Regarding mortality, previous studies have already demonstrated a linear association between serum urate levels and an increased risk of all-cause mortality and cardiovascular mortality, independent of common cardiovascular risk factors (10). These findings suggest that increasing the serum urate concentration could improve risk assessment in clinical practice by better discriminating and reclassifying individuals at higher risk of overall and cardiovascular mortality. An increased mortality risk is associated with both gouty and asymptomatic hyperuricemia (11, 12). However, the results varied considerably across these studies due to certain limitations, including notably small sample sizes, relatively short follow-up periods, and the inclusion of populations that may not fully represent the broader demographic spectrum.

Moreover, recent data suggest that the associations between urate levels or blood pressure and clinical events may be complex rather than straightforward linear correlations (13, 14). Very low urate levels might also confer an increased risk of adverse events or mortality (15–17). Elucidating the shape of these associations is crucial for determining thresholds for optimal urate levels, given that the efficacy and utility of urate-lowering therapy for cardiovascular protection remain debated (18, 19).

To compensate for the shortcomings of these previous studies, the present study was performed to prospectively investigate the association of serum urate levels with all-cause mortality in a nationally representative sample of American adult patients with hypertension. We also assessed potential nonlinear relationships of urate levels with hypertension and mortality.

## 2 Methods

### 2.1 Study design

This study is based on data obtained from the National Health and Nutrition Examination Survey (NHANES), an ongoing survey conducted by the National Center for Health Statistics (NCHS) in the United States. The NHANES serves as a vital tool for assessing the health and nutritional status of the noninstitutionalized population and plays a pivotal role in supplying crucial data for informing health policy, supporting research endeavors, and facilitating public health initiatives. Comprehensive details about the survey's design and data files can be accessed through the following link: https://www.cdc.gov/nchs/nhanes/ (accessed on August 31, 2023). To ensure ethical standards, the research protocol for this study received approval from the Research Ethics Review Board of the NCHS. Furthermore, it is important to note that NHANES collected informed written consent from all participants.

### 2.2 Population

In the present study, we included all participants aged more than 20 years (unweighted *n* = 97,657) from the NHANES cycles occurring between 2001 and 2018. Participants with missing data on urate levels, hypertension, or other comorbidities (heart failure, coronary artery disease, diabetes, stroke, cancer) were excluded. Additionally, those with ineligible data on mortality during follow-up were excluded. Our final analytic sample consisted of 38,644 participants.

### 2.3 Hyperuricemia, hypertension and mortality

Hyperuricemia was defined as a serum urate level >420 μmol/L (−7.0 mg/dl) in males and >360 μmol/L (−6.0 mg/dl) in females (20). Hypertension was identified based on participants' response to the following question: “Have you ever been told you have high blood pressure?”. To determine mortality status during the follow-up period, we utilized the NHANES public-use linked mortality file, last updated on April 26, 2022. This file is linked with the National Center for Health Statistics (NCHS) and the National Death Index (NDI) through a probability matching algorithm.

### 2.4 Ascertainment of covariates

We identified several variables as potential confounding factors in our analysis, covering various key aspects: (1) demographic characteristics: age (in years); sex (categorized as male or female); race/ethnicity (classified as Mexican American, other Hispanic, non-Hispanic white, non-Hispanic black, or other/multiracial); family poverty income ratio (PIR); education level (grouped as less than 9th grade, 9–11th grade, high school graduate, some college, or college graduate or above); and marital status (married/living with partner, widowed/divorced/separated, and never married). (2) Health status was assessed by the following measures: body mass index (BMI) (kg/m^2^), which was categorized as follows: underweight (<18.5), normal weight (<25), overweight (25–<30), and obese (≥30); waist circumference (cm); and smoking status (smoker or nonsmoker). (3) Whole-blood biochemical markers, including serum urate (μmol/L), total cholesterol (mmol/L), triglyceride (mmol/L), LDL-cholesterol (mmol/L), and creatinine (mg/dl), were measured in the NHANES Laboratory. (4) Patients' history of various health conditions was determined using self-reported doctor diagnoses: diabetes mellitus, stroke, cancer, heart failure, and coronary artery disease. (5) The estimated glomerular filtration rate (eGFR) was calculated based on the Epidemiology Collaboration CKD-EPI creatinine equation (21, 22): eGFR (ml/min/m^2^) = 141.0 × min (Scr/κ, 1)^α^ × max (Scr/κ, 1)^−1.209^ × 0.993^age^ × 1.018 (if woman) × 1.159 (if black). In this equation, Scr represent the standard serum creatinine (mg/dl). For women, use κ = 0.7, α = −0.329, and for men, use κ = 0.9, α = −0.411. Utilize the min function to calculate the minimum of Scr/κ or 1, and the max function to determine the maximum of Scr/κ or 1. Participants were categorized into non-chronic kidney disease group (eGFR ≥60 ml/min/m^2^) and chronic kidney disease group (eGFR <60 ml/min/m^2^) based on eGFR measures.

### 2.5 Statistical analysis

All estimates were weighted after taking the primary sampling unit, pseudostrata, and sampling weights to account for the complex sampling design unless otherwise specified. Participant characteristics were summarized using descriptive statistics. The prevalence of hyperuricemia was calculated, and participant demographics, clinical profiles, and comorbidities were compared by hyperuricemia status using t tests or Mann–Whitney *U*-tests for continuous variables and Wilcoxon rank-sum tests or chi-square tests for categorical variables. Associations between hyperuricemia and comorbidities were analyzed with multivariate logistic regression. Cox proportional hazard models were used to assess the relationship between hyperuricemia and mortality risk. Nonlinear associations between serum urate levels and hypertension and mortality were evaluated using restricted cubic spline analysis and visualized using spline curves. Kaplan‒Meier curves were calculated to display all-cause mortality among participants with and without hyperuricemia. All the statistical analyses were performed in R (version 4.3.1, R Foundation for Statistical Computing, Vienna, Austria) (“survey” packages in R account for the complex survey design were used). A two-sided *P*-value <0.05 was considered indicative of a statistically significant difference.

## 3 Results

### 3.1 Demographic and clinical characteristics

The characteristics of all the included participants are presented in Table 1. Among the 38,644 unweighted participants (corresponding to weighted 159,679,793 nationally representative participants), 6,956 (18.0%) had hyperuricemia, while 31,688 (82.0%) had nonhyperuricemia. Compared to participants without hyperuricemia, those with hyperuricemia were older (mean age 50.91 vs. 45.84 years, *p* < 0.001) and had a greater proportion of males (56.88% vs. 46.32%, *p* < 0.001). Participants with hyperuricemia also had a significantly greater mean BMI, waist circumference, blood pressure, total cholesterol, triglycerides, LDL cholesterol, and lower eGFR (all *p* < 0.001). The prevalence of obesity was markedly greater in the hyperuricemia group (55.20% vs. 30.35%, *p* < 0.001). With regard to race/ethnicity, Mexican Americans and non-Hispanic blacks comprised lower proportions of hyperuricemia cases than did non-Hispanic whites (*p* < 0.001). Those in the hyperuricemia group had lower educational attainment and were less likely to be married (*p* < 0.001). Approximately half of the individuals in the hyperuricemia group were current smokers, whereas 45.62% of those in the nonhyperuricemia group were current smokers (*p* < 0.001). The incidences of self-reported hypertension, diabetes, stroke, heart failure, coronary artery disease, cancer, and chronic kidney disease based on eGFR were all significantly greater among those with hyperuricemia (all *p* < 0.001).

**Table 1:** Demographic characteristics of all included participants with and without hyperuricemia.

Characteristic	Overall (*N* = 38,644)	Hyperuricemia (*N* = 6,956)	Non-hyperuricemia (*N* = 31,688)	*P*-value
Weighted participants (%)	159,679,793	27,658,230 (17.32%)	132,021,563 (82.68%)	
Age (year)	46.72 (16.80)	50.91 (17.72)	45.84 (16.46)	<0.001
Age group (%)				<0.001
20–39 years	12,942 (35.75%)	1,658 (28.43%)	11,284 (37.28%)	
40–59 years	12,019 (36.65%)	1,946 (33.98%)	10,073 (37.21%)	
60–79 years	9,739 (19.40%)	2,345 (26.18%)	7,394 (17.98%)	
≥80 years	3,944 (8.19%)	1,007 (11.41%)	2,937 (7.52%)	
Sex (%)				<0.001
Male	18,649 (48.15%)	3,856 (56.88%)	14,793 (46.32%)	
Female	19,995 (51.85%)	3,100 (43.12%)	16,895 (53.68%)	
Race/ethnicity (%)				<0.001
Mexican American	6,637 (8.22%)	829 (5.63%)	5,808 (8.77%)	
Other Hispanic	3,203 (5.19%)	437 (3.93%)	2,766 (5.45%)	
Non-Hispanic White	17,732 (69.25%)	3,380 (71.74%)	14,352 (68.72%)	
Non-Hispanic Black	7,759 (10.71%)	1,736 (12.38%)	6,023 (10.36%)	
Other/multiracial	3,313 (6.64%)	574 (6.33%)	2,739 (6.70%)	
Poverty income ratio	3.01 (1.64)	2.93 (1.61)	3.02 (1.64)	0.004
Educational attainment (%)				<0.001
Less than 9th grade	4,528 (5.99%)	808 (6.24%)	3,720 (5.94%)	
9–11th grade	5,661 (11.12%)	1,037 (11.35%)	4,624 (11.07%)	
High school graduate/GED	8,913 (23.25%)	1,752 (26.01%)	7,161 (22.67%)	
Some college or AA degree	11,042 (31.39%)	2,031 (32.71%)	9,011 (31.12%)	
College graduate or above	8,500 (28.24%)	1,328 (23.70%)	7,172 (29.20%)	
Marital status (%)				<0.001
Married/living with partner	23,510 (64.38%)	3,966 (61.41%)	19,544 (65%)	
Widowed/divorced/separated	8,371 (18.13%)	1,919 (22.16%)	6,452 (17.29%)	
Never married	6,763 (17.49%)	1,071 (16.43%)	5,692 (17.71%)	
Smoking status (%)				<0.001
Nonsmoker	20,975 (53.78%)	3,505 (50.92%)	17,470 (54.38%)	
Smoker	17,669 (46.22%)	3,451 (49.08%)	14,218 (45.62%)	
BMI (%)				<0.001
Obese (≥30)	13,593 (34.63%)	3,710 (55.20%)	9,883 (30.35%)	
Overweight (25–30)	12,959 (33.82%)	2,110 (31.37%)	10,849 (34.33%)	
Normal (18.5–25)	10,808 (29.91%)	925 (13.03%)	9,883 (33.42%)	
Underweight (<18.5)	609 (1.64%)	37 (0.40%)	572 (1.90%)	
Waist circumference (cm)	98.15 (16.09)	107.40 (15.99)	96.25 (15.44)	<0.001
Total cholesterol (mmol/L)	5.08 (1.09)	5.22 (1.14)	5.05 (1.08)	<0.001
Triglyceride (mmol/L)	1.51 (1.35)	1.92 (1.63)	1.42 (1.26)	<0.001
LDL-cholesterol (mmol/L)	2.98 (0.91)	3.08 (0.95)	2.96 (0.90)	<0.001
eGFR (ml/min/1.73 m^2^)	48.90 (50.12)	33.14 (41.59)	52.18 (51.11)	<0.001
Hypertension (%)				<0.001
Yes	13,092 (30.08%)	3,798 (49.34%)	9,294 (26.05%)	
No	25,552 (69.92%)	3,158 (50.66%)	22,394 (73.95%)	
Diabetes (%)				<0.001
Yes	4,624 (8.81%)	1,234 (13.66%)	3,390 (7.80%)	
No	34,020 (91.19%)	5,722 (86.34%)	28,298 (92.20%)	
Stroke (%)				<0.001
Yes	1,378 (2.67%)	440 (4.81%)	938 (2.22%)	
No	37,266 (97.33%)	6,516 (95.19%)	30,750 (97.78%)	
Cancer (%)				<0.001
Yes	3,478 (9.27%)	834 (11.69%)	2,644 (8.77%)	
No	35,166 (90.73%)	6,122 (88.31%)	29,044 (91.23%)	
Heart failure (%)				<0.001
Yes	1,205 (2.32%)	524 (5.73%)	681 (1.60%)	
No	37,439 (97.68%)	6,432 (94.27%)	31,007 (98.40%)	
Coronary artery disease (%)				<0.001
Yes	1,561 (3.31%)	476 (5.58%)	1,085 (2.83%)	
No	37,083 (96.69%)	6,480 (94.42%)	30,603 (97.17%)	
Chronic kidney disease (%)				<0.001
Yes	3,116 (6.97%)	1,484 (18.74%)	1,632 (4.52%)	
No	31,002 (93.03%)	4,648 (81.26%)	26,354 (95.48%)	

Continuous variables are presented as the means ± standard deviations; categorical variables are presented as numbers and percentages. Wilcoxon rank-sum test for complex survey samples; chi-square test with Rao & Scott's second-order correction. GED, general educational development.

### 3.2 Association of hyperuricemia with comorbidities

The associations between hyperuricemia and the presence of various comorbidities are summarized in Table 2. According to the unadjusted logistic regression analysis, participants with hyperuricemia had 2.19 times greater odds of hypertension (95% CI 2.03–2.37, *p* < 0.001) and 2.25 times greater odds of stroke (95% CI 1.87–2.71, *p* < 0.001); additionally, they had 1.36 times greater odds of heart failure (95% CI 1.16–1.60, *p* < 0.001), 2.45 times greater odds of obesity (95% CI 2.27–2.65, *p* < 0.001), and 4.39 times greater odds of chronic kidney disease (95% CI 3.91–4.93, *p* < 0.001) than did those without hyperuricemia. After we adjusted for age, sex, and smoking status, hyperuricemia was significantly associated with increased odds of hypertension (adjusted OR 2.04, 95% CI 1.88–2.22; *p* < 0.001), stroke (adjusted OR 2.18, 95% CI 1.80–2.64; *p* < 0.001), heart failure (adjusted OR 1.34, 95% CI 1.13–1.58; *p* < 0.001), obesity (adjusted OR 2.56, 95% CI 2.37–2.77; *p* < 0.001), and chronic kidney disease (adjusted OR 3.93, 95% CI 3.54–4.37; *p* < 0.001). No significant associations were detected between hyperuricemia and diabetes, cancer, or coronary artery disease according to the adjusted models (all *p* > 0.05).

**Table 2:** Weighted logistic regression demonstrating the relationship between hyperuricemia and the presence of comorbidities.

Comorbidity	Unadjusted association			Adjusted association^a^		
	OR	95% CI	*P*-value	OR	95% CI	*P*-value
Hypertension	2.19	2.03, 2.37	<0.001	2.04	1.88, 2.22	<0.001
Diabetes	0.96	0.85, 1.07	0.4	0.90	0.80, 1.01	0.079
Stroke	2.25	1.87, 2.71	<0.001	2.18	1.80, 2.64	<0.001
Cancer	1.07	0.88, 1.29	0.5	0.89	0.73, 1.09	0.3
Heart failure	1.36	1.16, 1.60	<0.001	1.34	1.13, 1.58	<0.001
Coronary artery disease	1.10	0.98, 1.23	0.10	1.02	0.91, 1.15	0.7
Obesity	2.45	2.27, 2.65	<0.001	2.56	2.37, 2.77	<0.001
Chronic kidney disease	4.39	3.91, 4.93	<0.001	3.93	3.54, 4.37	<0.001

OR, odds ratio; CI, confidence interval.

^a^ Adjusted by age, sex, and smoking status.

### 3.3 Characteristics of hypertension

A comparison of clinical characteristics between hyperuricemic patients with and without hypertension is provided in Table 3. Among the 6,956 patients with hyperuricemia, 3,798 (54.58%) had hypertension, and 3,158 (45.38%) did not have hypertension. Those with hypertension were older than were those without hypertension (mean age 58.84 vs. 43.18 years, *p* < 0.001). A lower proportion of hypertensive patients were male than nonhypertensive patients were (46.47% vs. 67.00%, *p* < 0.001). The racial distribution differed between the groups, with non-Hispanic blacks accounting for a greater percentage of hypertensive patients (15.35% vs. 9.48%, *p* < 0.001). Hypertensive participants had lower educational attainment and were more likely to be widowed/divorced/separated than non-hypertensive participants were (*p* < 0.001 for both). Over half of the hypertensive patients were obese (60.58%), whereas 50.02% of the nonhypertensive patients were obese (*p* < 0.001). The mean BMI, waist circumference, triglyceride level, and urate level were also greater in patients with hypertension (*p* < 0.001 for BMI and waist circumference; *p* < 0.05 for other measures). The incidences of diabetes, stroke, cancer, heart failure, coronary artery disease, and chronic kidney disease were all significantly greater in the hypertensive group (all *p* < 0.001).

**Table 3:** Demographic and clinical characteristics of hyperuricemic patients according to hypertension status.

Characteristic	Overall (*N* = 6,956)	Hypertension (*N* = 3,798)	Non-hypertension (*N* = 3,158)	*P*-value
Weighted participants (%)	27,658,230	13,645,504 (49.34%)	14,012,726 (50.66%)	
Age (year)	50.91 (17.72)	58.84 (15.20)	43.18 (16.55)	<0.001
Age group				<0.001
60–79 years	2,345 (26.18%)	1,769 (38.77%)	576 (13.91%)	
40–59 years	1,946 (33.98%)	1,001 (34.31%)	945 (33.66%)	
20–39 years	1,658 (28.43%)	330 (11.19%)	1,328 (45.23%)	
≥80 years	1,007 (11.41%)	698 (15.73%)	309 (7.20%)	
Male (%)				<0.001
Male	3,856 (56.88%)	1,788 (46.47%)	2,068 (67.00%)	
Female	3,100 (43.12%)	2,010 (53.53%)	1,090 (33.00%)	
Race/ethnicity				<0.001
Mexican American	829 (5.63%)	364 (3.59%)	465 (7.61%)	
Other Hispanic	437 (3.93%)	210 (2.78%)	227 (5.05%)	
Non-Hispanic White	3,380 (71.74%)	1,852 (72.97%)	1,528 (70.55%)	
Non-Hispanic Black	1,736 (12.38%)	1,138 (15.35%)	598 (9.48%)	
Other/multiracial	574 (6.33%)	234 (5.31%)	340 (7.32%)	
Poverty income ratio	2.93 (1.61)	2.90 (1.59)	2.97 (1.63)	0.2
Educational attainment				<0.001
Less than 9th grade	808 (6.24%)	502 (7.46%)	306 (5.05%)	
9–11th grade	1,037 (11.35%)	617 (12.61%)	420 (10.13%)	
High school graduate/GED	1,752 (26.01%)	964 (25.85%)	788 (26.15%)	
Some college or AA degree	2,031 (32.71%)	1,080 (32.96%)	951 (32.46%)	
College graduate or above	1,328 (23.70%)	635 (21.12%)	693 (26.21%)	
Marital status				<0.001
Married/living with partner	3,966 (61.41%)	2,130 (62.21%)	1,836 (60.63%)	
Widowed/divorced/separated	1,919 (22.16%)	1,326 (29.14%)	593 (15.36%)	
Never married	1,071 (16.43%)	342 (8.65%)	729 (24.01%)	
Smoking status				0.003
Nonsmoker	3,505 (50.92%)	1,837 (48.70%)	1,668 (53.07%)	
Smoker	3,451 (49.08%)	1,961 (51.30%)	1,490 (46.93%)	
BMI				<0.001
Obese (≥30)	3,710 (55.20%)	2,174 (60.58%)	1,536 (50.02%)	
Overweight (25–30)	2,110 (31.37%)	1,064 (28.69%)	1,046 (33.95%)	
Normal (18.5–25)	925 (13.03%)	429 (10.34%)	496 (15.62%)	
Underweight (<18.5)	37 (0.40%)	19 (0.39%)	18 (0.41%)	
Waist circumference (cm)	107.40 (15.99)	109.99 (15.85)	104.95 (15.74)	<0.001
Total cholesterol (mmol/L)	5.22 (1.14)	5.19 (1.17)	5.26 (1.10)	0.021
Triglyceride (mmol/L)	1.92 (1.63)	1.97 (1.48)	1.87 (1.76)	0.016
LDL-cholesterol (mmol/L)	3.08 (0.95)	2.98 (0.96)	3.18 (0.92)	<0.001
Serum urate (μmol/L)	445.44 (56.82)	448.38 (61.83)	442.58 (51.33)	0.1
eGFR (ml/min/1.73 m^2^)	33.14 (41.59)	36.97 (39.03)	29.27 (43.69)	<0.001
Diabetes (%)				<0.001
Yes	1,234 (13.66%)	1,013 (22.59%)	221 (4.96%)	
No	5,722 (86.34%)	2,785 (77.41%)	2,937 (95.04%)	
Stroke (%)				<0.001
Yes	440 (4.81%)	363 (7.99%)	77 (1.70%)	
No	6,516 (95.19%)	3,435 (92.01%)	3,081 (98.30%)	
Cancer (%)				<0.001
Yes	834 (11.69%)	634 (17.07%)	200 (6.45%)	
No	6,122 (88.31%)	3,164 (82.93%)	2,958 (93.55%)	
Heart failure (%)				<0.001
Yes	524 (5.73%)	440 (9.56%)	84 (2.00%)	
No	6,432 (94.27%)	3,358 (90.44%)	3,074 (98.00%)	
Coronary artery disease (%)				<0.001
Yes	476 (5.58%)	386 (8.96%)	90 (2.29%)	
No	6,480 (94.42%)	3,412 (91.04%)	3,068 (97.71%)	
Chronic kidney disease				<0.001
Yes	1,484 (18.74%)	1,186 (28.87%)	298 (8.48%)	
No	4,648 (81.26%)	2,208 (71.13%)	2,440 (91.52%)	

Continuous variables are presented as the means ± standard deviations; categorical variables are presented as numbers and percentages. Wilcoxon rank-sum test for complex survey samples; chi-square test with Rao & Scott's second-order correction. GED, general educational development.

### 3.4 Risk factors for hypertension

The risk factors for hypertension among individuals with hyperuricemia are displayed in Table 4. Significantly greater odds of having hypertension were associated with older age, female sex, non-Hispanic black race, diabetes, obesity, stroke, cancer, and heart failure. Specifically, each one-year increase in age was associated with 5% greater odds of having hypertension (OR 1.05, 95% CI 1.04–1.06; *p* < 0.001). Compared with males, females had 34% greater odds of having hypertension (OR 1.34, 95% CI 1.16–1.56; *p* < 0.001). Compared to Mexican Americans, individuals of other races/ethnicities had greater odds of having hypertension (*p* < 0.001). Diabetes was linked to 2.66-fold greater odds (95% CI 2.11–3.37, *p* < 0.001), and obesity was linked to 1.75-fold greater odds (95% CI 1.48–2.08, *p* < 0.001). Stroke, cancer, and heart failure were associated with 1.72, 1.45-, and 1.46-fold greater odds, respectively (*p* < 0.01 for all). The marital status of “never married” was linked to 31% lower odds of having hypertension than “married” status was (adjusted OR 0.69, 95% CI 0.54–0.87; *p* = 0.002).

**Table 4:** The risk factors for concomitant hypertension in participants with hyperuricemia.

Characteristic	OR	95% CI	*P*-value
Age	1.05	1.04, 1.06	<0.001
Female	1.34	1.16, 1.56	<0.001
Race/ethnicity			
Mexican American	–	–	<0.001
Other Hispanic	1.07	0.76, 1.50	0.693
Non-Hispanic White	1.44	1.14, 1.83	0.003
Non-Hispanic Black	2.53	1.99, 3.21	<0.001
Other/multiracial	1.38	0.97, 1.96	0.070
Marital status			
Married/living with partner	–	–	0.004
Widowed/divorced/separated	0.98	0.79, 1.21	0.834
Never married	0.69	0.54, 0.87	0.002
Educational attainment			
Less than 9th grade	–	–	0.2
9–11th grade	1.07	0.78, 1.46	0.693
High school graduate/GED	0.92	0.69, 1.22	0.548
Some college or AA degree	1.03	0.77, 1.38	0.824
College graduate or above	0.83	0.61, 1.14	0.248
Poverty income ratio	1.02	0.96, 1.08	0.5
Smoking status			
Nonsmoker	–	–	0.4
Smoker	1.07	0.92, 1.25	0.392
Diabetes	2.66	2.11, 3.37	<0.001
Heart failure	1.46	0.96, 2.23	0.073
Coronary artery disease	1.44	0.98, 2.13	0.063
Stroke	1.72	1.15, 2.59	0.008
Cancer	1.45	1.15, 1.83	0.002
Obesity	1.75	1.48, 2.08	<0.001
Chronic kidney disease	1.18	0.93, 1.49	0.2

OR, odds ratio; CI, confidence interval.

### 3.5 Mortality rates according to hyperuricemia and hypertension status

The all-cause and cardiovascular mortality rates in patients with different combinations of hyperuricemia and hypertension are summarized in Table 5. According to adjusted Cox proportional hazards models, compared with no hyperuricemia, hyperuricemia alone was associated with a 1.48- and 1.95-fold greater hazard of all-cause and cardiovascular mortality, respectively. Similarly, hypertension alone conferred a 1.42- and 1.69-fold greater mortality hazard than did non-hypertension. The highest mortality risk was observed in participants with both hyperuricemia and hypertension. This group had a 1.87- and 2.82-fold greater hazard of death than did the non-hyperuricemic participants without hypertension.

**Table 5:** All-cause and cardiovascular mortality according to hypertension and hyperuricemia status.

Characteristic	All-cause mortality			Cardiovascular mortality		
	HR	95% CI	*P*-value	HR	95% CI	*P*-value
Non-hyperuricemia (ref)						
Hyperuricemia	1.48	1.34, 1.62	<0.001	1.95	1.59, 2.38	<0.001
Non-hypertension (ref)						
Hypertension	1.42	1.29, 1.56	<0.001	1.69	1.36, 2.09	<0.001
Non-hyperuricemia and non-hypertension (ref)						
Non-hyperuricemia and hypertension	1.34	1.18, 1.52	<0.001	1.65	1.29, 2.12	<0.001
Hyperuricemia and non-hypertension	1.38	1.18, 1.63	<0.001	2.09	1.46, 2.99	<0.001
Hyperuricemia and hypertension	1.87	1.64, 2.13	<0.001	2.82	2.11, 3.77	<0.001

Adjusted by age, sex, smoking status and BMI. HR, hazard ratio; CI, confidence interval.

### 3.6 Nonlinear relationships of serum urate levels and hypertension with mortality

**Figure 1:**
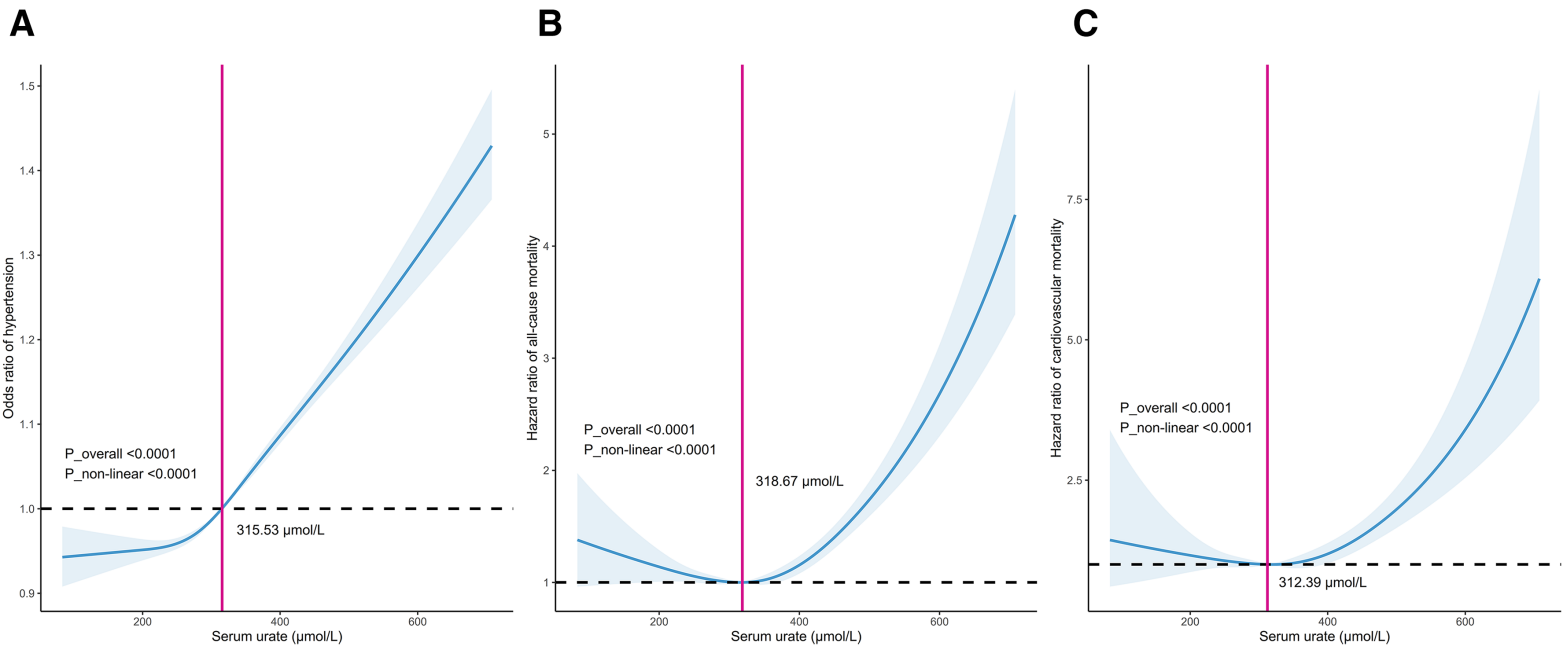
Dose–response relationship between serum urate levels and the incidence of hypertension (**A**), long-term mortality including all-cause mortality (**B**) and cardiovascular mortality (**C**).

In all included participants, a nonlinear J-shaped association was found between serum urate levels and hypertension risk, even after adjusting for multiple covariates, as evidenced by RCS analysis (*P*_overall <0.001). Notably, there was an inflection point at a urate level of 315.53 μmol/L. Below this cutoff value, the risk of hypertension steadily decreased as urate levels decreased. Conversely, above this threshold, the risk of hypertension progressively increased in a dose-dependent manner with increasing urate levels (*P*_nonlinear <0.001) (Figure 1A).

In patients with hypertension, there was a distinct U-shaped association between serum urate levels and the risk of all-cause and cardiovascular mortality (both *P*_overall <0.001). A discernible threshold effect emerged, marked by an inflection point at urate levels of 313.46 μmol/L and 312.39 μmol/L. Below these thresholds, mortality risk consistently increased as urate levels decreased, while above this critical point, mortality risk exhibited an upward trajectory once more with escalating urate levels (*P*_nonlinear <0.001) (Figures 1B,C).

### 3.7 Kaplan–Meier survival curves

**Figure 2:**
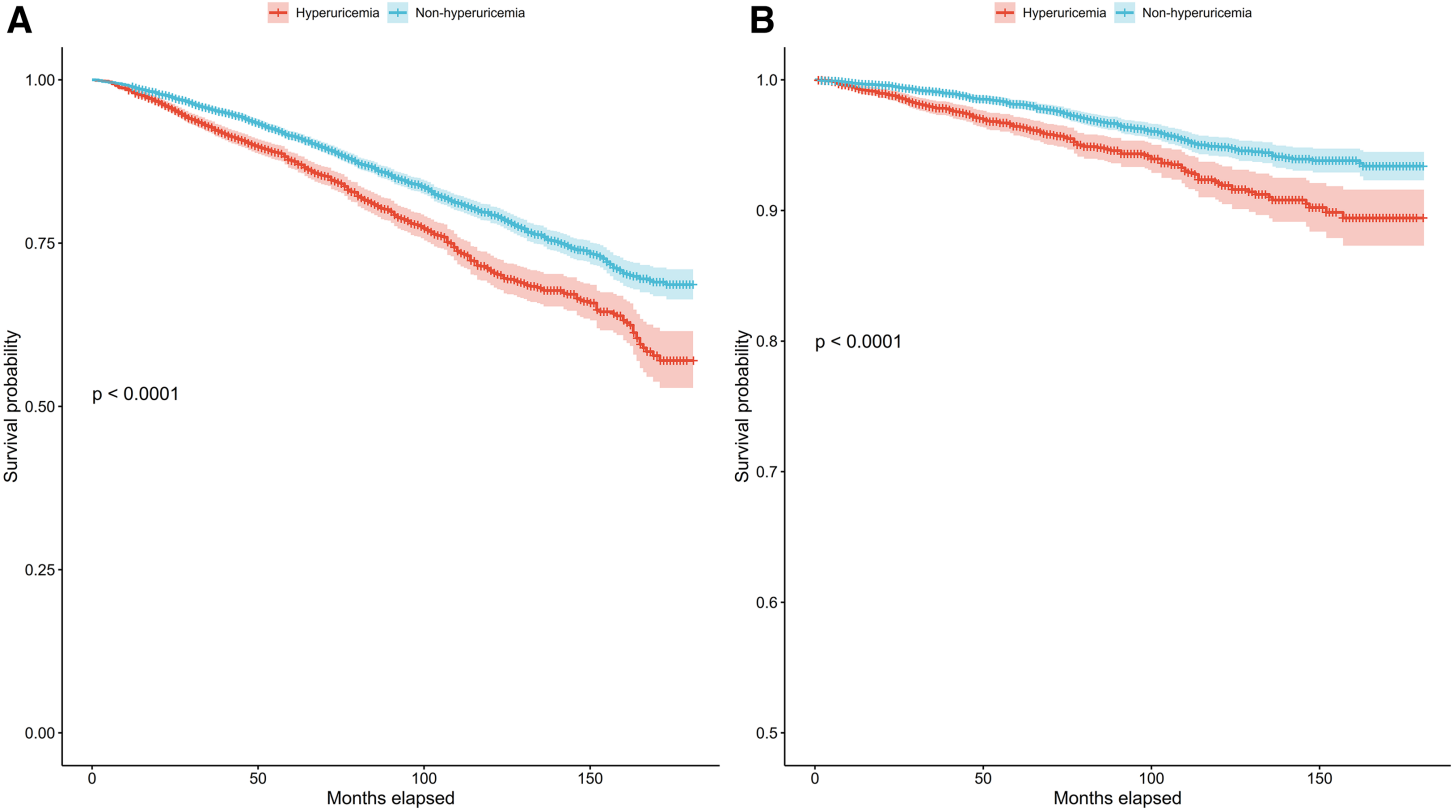
Kaplan–Meier survival curves for the hyperuricemia and non-hyperuricemia groups for all-cause mortality (**A**) and cardiovascular mortality (**B**) in patients with hypertension.

In patients with hypertension, we also conducted Kaplan‒Meier survival analysis to evaluate the correlation between survival time and survival probability at different serum urate levels. The median follow-up period was 78 months for all patients with hyperuricemia. The results showed significantly greater mortality among those with hyperuricemia than among those without hyperuricemia for both all-cause and cardiovascular mortality (Figures 2A,B) (*p* for log-rank test <0.001).

## 4 Discussion

In this study, 17.3% of the hyperuricemic population was diagnosed midway within the global range of 13%–25% noted in prior studies (23). The prevalence of hyperuricemia is related to age, sex, race/ethnicity, and lifestyle factors. The likelihood of elevated urate increases with age up to the fifth decade of life. We discovered that hyperuricemia was strongly associated with hypertension, even after adjusting for other comorbidities, such as stroke, heart failure, and obesity. In alignment with prior research, hyperuricemia is more common in men than in premenopausal women, likely due to the uricosuric effects of estrogen (24, 25). However, it is intriguing that once women develop hyperuricemia, the incidence of hypertension significantly surpasses that of men, with rates of 53.53% for women compared to 33.00% for men, which is also consistent with previous reports (26). This association underscores the relevance of hyperuricemia as a potential risk factor for hypertension. Importantly, we observed that both hyperuricemia and hypertension were independently linked to an increased risk of all-cause mortality, with adjusted hazard ratios (HRs) of 1.48 and 1.42, respectively. The highest mortality risk was observed in individuals who presented with both conditions, indicating a synergistic effect (HR 1.87). The restricted cubic spline model showed nonlinear correlations between serum urate concentrations and hypertension (J-shaped) and between serum urate concentrations and all-cause mortality (U-shaped). These findings underscore the significance of considering both hyperuricemia and hypertension as independent risk factors for all-cause mortality and emphasize the need for a more nuanced understanding of their interplay.

The role of serum urate in hypertension development remains a subject of ongoing research and is not yet fully understood. Several proposed mechanisms have been identified. Urate can activate various oxidative stress pathways involving xanthine oxidase, nicotinamide adenine dinucleotide phosphate (NADPH) oxidase, the renin-angiotensin-aldosterone system (RAAS), and inflammatory cytokines. This oxidative stress damages blood vessels and contributes to hypertension (27, 28). Hyperuricemia is also closely associated with obesity and can trigger systemic inflammation and activate the RAAS and sympathetic nervous systems (29, 30). It appears that urate may act as an endogenous danger signal, triggering the innate immune response, which is well established for its significant role in arterial hypertension (31). Although the exact underlying mechanisms are not fully understood, these primary pathways offer biologically plausible explanations for how elevated urate may directly contribute to the development of hypertension rather than merely having a passive association.

Prospective studies have played a crucial role in establishing the temporal relationship between hyperuricemia and hypertension. These studies follow individuals without hypertension at baseline and track the development of hypertension over several years. The findings consistently show that individuals with higher baseline urate levels are at greater risk of developing hypertension (9, 32). Many studies have further reported a dose‒response relationship, wherein a higher level of serum urate is associated with a progressively greater risk of hypertension. This gradient of risk strengthens the argument for a causal relationship between hyperuricemia and hypertension (33). Additionally, clinical trials have investigated the impact of urate-lowering therapies on blood pressure. A cohort study involving 100 participants with asymptomatic hyperuricemia revealed that, compared with HCs, those receiving allopurinol experienced significant reductions in office systolic and diastolic blood pressure, central systolic blood pressure, pulse pressure, carotid intima-media thickness, and hs-CRP. These improvements are indicative of a positive impact on a patient's long-term prognosis (34). Furthermore, a meta-analysis of seven eligible trials involving 503 participants with hyperuricemia demonstrated that urate-lowering therapy led to decreases in systolic and diastolic blood pressure, fasting insulin levels, and homeostasis model assessment of insulin resistance (35). However, it is worth noting that not all trials have shown consistent results, as some failed to provide sufficient evidence to support an effect on blood pressure, proteinuria, or other cardiovascular markers through urate-lowering therapy (36).

Regarding the role of hyperuricemia in mortality, a growing body of epidemiological evidence indicates that hyperuricemia is independently associated with higher all-cause mortality (37). A recent systematic review and meta-analysis involving 14 studies and more than 341,389 adults revealed a 20% increased risk of all-cause mortality associated with hyperuricemia in general populations (38). This heightened risk of mortality seems to be applicable to both gout patients and asymptomatic individuals with elevated urate levels (24). The link between hyperuricemia and increased all-cause mortality likely involves various pathophysiological mechanisms. As previously discussed in the context of hypertension, elevated urate levels can lead to endothelial dysfunction, vascular inflammation, oxidative stress, insulin resistance, and systemic metabolic abnormalities. These factors contribute to the development and progression of various chronic diseases, including cardiovascular diseases, renal dysfunction, and metabolic disorders, all of which can further increase the risk of mortality (39–41). Our findings of heightened all-cause and cardiovascular mortality in patients with hyperuricemia and hypertension agree with those of several previous studies (38, 42, 43).

Notably, our findings revealed a U-shaped pattern of nonlinear relationships between serum urate levels and mortality risk, indicating that mortality risk consistently increased again as urate levels decreased below the inflection point. This result is consistent with previous studies using large samples of individuals in the general population (44, 45) and those assessing patients with cardiovascular diseases (46–48). Although the exact reasons for the heightened mortality risk associated with low levels of serum urate remain unclear, several potential explanations exist. First, low serum urate levels might merely reflect malnutrition status, cachexia, and other wasting conditions (49). Additionally, urate has antioxidant effects, such as the scavenging of free radicals, which are unstable molecules that can cause damage to cells (50). Urate also binds to iron ions, preventing them from catalyzing oxidative reactions that generate free radicals (51).

Moreover, it is conceivable that the observed low levels of serum urate may be partially attributable to the administration of medications designed to reduce serum urate levels. For example, the findings from an Austrian study that surveyed a broad population indicate that individuals who begin treatment with febuxostat face a greater likelihood of experiencing nonfatal cardiovascular events or mortality from any cause than do those treated with allopurinol (52). However, in a cohort of 99,744 elderly gout patients, febuxostat initiation did not increase cardiovascular or mortality risk compared to allopurinol, although a slight long-term mortality trend and a reduced heart failure exacerbation risk were noted with febuxostat use (53). This underlying factor could influence mortality rates in individuals exhibiting low serum urate levels.

Conflicting evidence exists regarding the relationship between urate lowering drugs and hypertension. Animal models suggest that urate induces hypertension via a two-step process: initial activation of the renin-angiotensin system and inhibition of nitric oxide, causing increased systemic vascular resistance, followed by urate-mediated vasculopathy of the renal afferent arterioles, resulting in late sodium-sensitive hypertension (54). However, urate reduction cannot yet be recommended as first-line hypertension therapy due to heterogeneous effects on blood pressure across studies for both pharmacological urate-lowering drugs and nonpharmacological measures such as dietary changes to reduce sugar, fructose and salt intake. Some studies have shown no effect of these interventions on blood pressure, while others have demonstrated blood pressure reduction (55, 56). However, further research is still needed to clarify the causal role of urate and its therapeutic implications in hypertension.

In this study, we identified cutoffs of 318.67 μmol/L (5.36 mg/dl) for all-cause mortality and 312.39 μmol/L (5.25 mg/dl) for cardiovascular mortality. These thresholds differed slightly from those reported in the URRAH series, conducted in middle-aged patients, where cutoffs of 4.7–to 6.8 mg/dl for all-cause mortality and 4.89–5.6 mg/dl for cardiovascular mortality were suggested (15). The variations in cutoff values are primarily dependent on comorbidities such as heart failure, diabetes, and kidney disease in the URRAH studies (17, 57). These differences highlight that the serum urate thresholds associated with adverse outcomes are likely influenced by factors like age and comorbidities. For now, the serum urate cutoffs for mortality risk remain inconclusive and should be interpreted in the context of the population studied.

This study has several limitations that should be acknowledged. First, the cross-sectional nature of the NHANES data limits causal inferences about the relationship between hyperuricemia and hypertension or mortality. The use of an observational design meant that unmeasured confounders could not be completely excluded. Additionally, hyperuricemia and hypertension were identified based on questionnaire administration rather than repeated measurements, which may have led to misclassification of some participants. However, information on the use of medications, especially urate-elevating agents such as diuretics, was not comprehensively available in our study. Some drug classes, such as diuretics, are known to have hyperuricemic effects and could partially explain the greater incidence of hyperuricemia in hypertensive patients than in normotensive individuals. Therefore, further longitudinal cohort studies are still needed to validate the findings from the present study.

## 5 Conclusion

In summary, the accumulating evidence suggests that elevated urate levels may be associated with an increased risk of all-cause and cardiovascular mortality in hypertensive patients. The nadirs we observed for optimal risk reduction may provide a rationale for targeting hyperuricemia for mortality prevention, particularly in higher-risk groups with cardiovascular diseases, metabolic abnormalities or multiple comorbidities. However, definitive interventional trials are still needed to determine whether lowering urate levels can improve survival.

## Data Availability

The original contributions presented in the study are included in the article/Supplementary Material, further inquiries can be directed to the corresponding author.
